# Electrochemical Glue for Binding Chitosan–Alginate Hydrogel Fibers for Cell Culture

**DOI:** 10.3390/mi13030420

**Published:** 2022-03-08

**Authors:** Yoshinobu Utagawa, Kosuke Ino, Tatsuki Kumagai, Kaoru Hiramoto, Masahiro Takinoue, Yuji Nashimoto, Hitoshi Shiku

**Affiliations:** 1 Graduate School of Environmental Studies, Tohoku University, Sendai 980-8579, Japan; yoshinobu.utagawa.p3@dc.tohoku.ac.jp (Y.U.); tatsuki.kumagai.1@gmail.com (T.K.); kaoru.hiramoto.s5@dc.tohoku.ac.jp (K.H.); 2 Graduate School of Engineering, Tohoku University, Sendai 980-8579, Japan; yuji.nashimoto.d8@tohoku.ac.jp; 3 Department of Computer Science, Tokyo Institute of Technology, Yokohama 226-8502, Japan; takinoue@c.titech.ac.jp; 4 Frontier Research Institute for Interdisciplinary Sciences, Tohoku University, Sendai 980-8578, Japan

**Keywords:** electrochemical glue, chitosan hydrogel, alginate hydrogel, interfacial polyelectrolyte complexation fiber

## Abstract

Three-dimensional organs and tissues can be constructed using hydrogels as support matrices for cells. For the assembly of these gels, chemical and physical reactions that induce gluing should be induced locally in target areas without causing cell damage. Herein, we present a novel electrochemical strategy for gluing hydrogel fibers. In this strategy, a microelectrode electrochemically generated HClO or Ca^2+^, and these chemicals were used to crosslink chitosan–alginate fibers fabricated using interfacial polyelectrolyte complexation. Further, human umbilical vein endothelial cells were incorporated into the fibers, and two such fibers were glued together to construct “+”-shaped hydrogels. After gluing, the hydrogels were embedded in Matrigel and cultured for several days. The cells spread and proliferated along the fibers, indicating that the electrochemical glue was not toxic toward the cells. This is the first report on the use of electrochemical glue for the assembly of hydrogel pieces containing cells. Based on our results, the electrochemical gluing method has promising applications in tissue engineering and the development of organs on a chip.

## 1. Introduction

Hydrogels have received increased attention for bioapplications because of their useful properties, such as their high water content, high permeability, biocompatibility, and shape retention. Therefore, hydrogels have been widely used for the biofabrication of three-dimensional (3D) tissue models, and 3D bioprinters have been used for the fabrication of complex-shaped hydrogels [1]. In this approach, hydrogels containing cells are extruded from a single syringe, and the syringe is moved programmatically using an XYZ stage. In another approach, small units of hydrogels, such as fibers, beads, sheets, and blocks, are fabricated using microfluidic systems [2,3] and photolithographic technologies [4,5]. After fabrication, these units are assembled to construct more complex-shaped hydrogels. Among these units, hydrogel fibers have been widely investigated because 3D hydrogels can be prepared by knitting the hydrogel fibers [6]. To date, several methods have been proposed for fabricating hydrogel fibers, such as the microfluidic method [6] and the use of sacrificial sugar templates [7]. Hydrogel fibers have also been fabricated using interfacial polyelectrolyte complexation (IPC) [8,9,10,11] and utilized for cell culture [12].

The ordered assembly of hydrogels is a promising method for building 3D hydrogel constructs. However, assembly techniques using glue are required to combine the hydrogel units. To date, several such techniques have been reported for hydrogel bonding, such as host–guest [13], electrostatic [14], and hydrogen-bonding interactions [15], as well as the use of catechol-based compounds [16], polysaccharides [17], stimuli-responsive curable polymers [18], and fibrin glue [19]. In addition, the use of pH-triggered bioinspired glues has been reported for hydrogel assembly [20]. In these processes, chemicals must be added or generated locally and on-demand to achieve local hydrogel assembly during cell culture. To achieve this simply, a chemical solution can be added to the hydrogel pieces using a micropipette for assembly, but local addition is difficult and the chemicals often affect cell viability. As an alternative approach, UV irradiation can be used with a photomask to induce local chemical reactions, but a photoinitiator is required, which also affects cell viability. To address this issue, we have developed a novel strategy to achieve gluing based on electrochemical reactions.

We and other groups have previously reported the electrochemical biofabrication of hydrogels [21,22,23,24,25,26,27,28,29,30,31,32,33,34,35,36,37,38,39,40]. For example, reactive oxidizing agents (e.g., HClO) have been electrochemically generated at an electrode in solution for crosslinking chitosan [23,41], resulting in the formation of chitosan hydrogels on the electrode. Electrochemically generated chemicals have also been utilized to modify cell culture surfaces locally using a scanning electrochemical microscope [42,43,44], resulting in cell patterning during culture. In another study, catechol was electrochemically patterned on hydrogels [45]. An electrodeposition method was applied for the fabrication of porous Janus films [39]. Electrodeposited hydrogels were also used as gel probes in scanning gel electrochemical microscopy [32]. In addition to this strategy, electrochemically generated Ca^2+^ has been utilized for the fabrication of Ca-alginate [26,27,30,33,34,38]. In this method, water electrolysis is induced to produce H^+^ at the anode, and Ca^2+^ is released from CaCO_3_ particles dispersed in a sodium alginate solution because of the corresponding acidification. Using this method, tube-shaped hydrogels can be fabricated using a wire electrode [27,33,37]. Additionally, 3D hydrogel constructs can be fabricated by using a 3D bioprinting system [34].

In these previous studies, electrochemically generated chemicals were used for the gelation of a solution containing biopolymers. In the present study, these electrochemically generated chemicals were used as the glue for assembling hydrogel pieces, and are thus, denoted electrochemical glue. Specifically, IPC chitosan or alginate hydrogel fibers were used because electrochemically generated chemicals, such as the reactive oxidizing agent and Ca^2+^, can interact with chitosan and alginate, respectively. As a simple demonstration, “+”-shaped hydrogels were fabricated by the adhesion of crossing points of two hydrogel fibers. In addition, because hydrogel fibers are widely used for the fabrication of vascular models, human umbilical vein endothelial cells (HUVECs) were incorporated into the fibers. The hydrogel fibers were cultured to investigate the effects of the electrochemical glue on the cell culture. Thus, this study is a proof of concept of the use of electrochemical glue for binding hydrogel pieces. In the future, this strategy could be used for the fabrication of 3D tissues and organs in regenerative medicine and drug screening.

## 2. Materials and Methods

### 2.1. Fabrication of Hydrogel Fibers

A 0.5 wt% sodium alginate (FUJIFILM Wako Pure Chemicals, Osaka, Japan) solution was prepared in deionized water. Chitosan powder (Sigma–Aldrich, St. Louis, MO, USA) was dissolved in acetate solution (pH 5.8) to prepare a 0.5 wt% chitosan solution. Then, droplets of 100 µL of sodium alginate and chitosan solutions were placed on a hydrophobic film. These droplets were brought into contact with each other using a 200-µL pipette tip to form an IPC film at the droplet–droplet interface. Then, the IPC film was drawn out by the upward motion of the pipette tip, resulting in the collection of the IPC fibers; that is, alginate–chitosan hydrogel fibers.

### 2.2. Electrochemical Gluing Induced by HClO

Two hydrogel fibers were orthogonally arranged in Dulbecco’s phosphate-buffered saline (PBS(-), Nacalai Tesque, Kyoto, Japan) containing KCl (FUJIFILM Wako Pure Chemicals, Japan) at various concentrations. The final concentration of Cl^−^ was adjusted to 0.15–0.50 M. A 50-µm-diameter Au disk microelectrode was used as the working electrode, and Ag/AgCl (sat. KCl) reference and Pt counter electrodes were inserted into the solution. These electrodes were connected to a potentiostat (HA1010mSM8, Hokuto Denko, Kyoto, Japan). The microelectrode was then placed near the crossing point of the fibers, and a voltage of 0.95 V was applied for 1–15 min to produce HClO near the electrode [41]. On exposure to HClO, the chitosan was partially oxidized to form aldehydes and then crosslinked via the formation of Schiff base linkages.

### 2.3. Electrochemical Gluing Induced by Ca^2+^

For electrochemical gluing induced by Ca^2+^, 5 mg/mL CaCO_3_ particles (FUJIFILM Wako Pure Chemicals, Japan) were dispersed in a 0.5 wt% sodium alginate solution. This solution and a 0.5 wt% chitosan solution were used for fiber fabrication. The resulting IPC fibers were immersed in the 0.5% sodium alginate solution for a few seconds.

Before electrochemical gluing using Ca^2+^, the effect of Ca^2+^ on the assembly of the hydrogel fibers was investigated. Two hydrogel fibers without CaCO_3_ particles were arranged orthogonally in PBS, and 100 μL of 100 mM CaCl_2_ solution was injected at the crossing points. To confirm the crosslinking, one of the fibers was physically pulled. To control the chemical glue, 100 μL of 100 mM HCl solution was injected at the crossing point of the IPC fibers containing CaCO_3_ particles. Immediately after addition, one of the fibers was pulled to check the crosslinking.

For electrochemical gluing via Ca^2+^, a Pt wire with a diameter of 300 µm or a Pt disk microelectrode with a diameter of 50 μm was used as the working electrode. The microelectrode was placed near the crossing point of the fibers in a solution and 1.8 V was then applied to produce H^+^ for 1 min, then Ca^2+^ was released near the electrode. To check the binding strength, after gluing, 10 mM ethylenediamine tetraacetic acid (EDTA) solution was injected into the glued area and the fibers were pulled.

### 2.4. Cell Culture

Green fluorescent protein (GFP)-expressing HUVECs (GFP-HUVECs, Angio-Proteomie, Boston, MA, USA) were cultured in endothelial growth medium (endothelial cell growth medium 2, Promo Cell, Heidelberg, Germany) containing 1% penicillin/streptomycin (PS, Gibco, Shanghai, China). MCF-7 cells (Institute of Development, Aging and Cancer, Tohoku University, Japan) were cultured in RPMI1640 (Gibco, China) containing 10% fetal bovine serum (FBS, Gibco, China) and 1% PS. All cells were maintained at 37 °C in a humidified atmosphere containing 5% CO_2_. Phase-contrast and fluorescence images of the cells were captured using a microscope (Olympus IX71, Tokyo, Japan).

### 2.5. Electrochemical Gluing Induced by Ca^2+^ for Binding of Hydrogel Fibers Containing HUVECs

Before the electrochemical glue was used for cell culture applications, the effects of the IPC fibers on cell growth were investigated using MCF-7 cells. Briefly, 5.0 × 10^6^ cells/mL MCF-7 cells were suspended in 0.5 wt% sodium alginate solution, and hydrogel fibers containing MCF-7 cells were fabricated. The fibers were then embedded in Matrigel (Product # 354234, Corning, New York, NY, USA) and cultured.

In addition, HUVECs were suspended in a 0.5 wt% sodium alginate solution containing 5 mg/mL CaCO_3_ particles, and the solution was used to prepare hydrogel fibers containing HUVECs. After electrochemical gluing, the glued fibers were cultured in a culture medium or Matrigel.

## 3. Results and Discussion

### 3.1. Fabrication of Hydrogel Fibers

**Figure 1:**
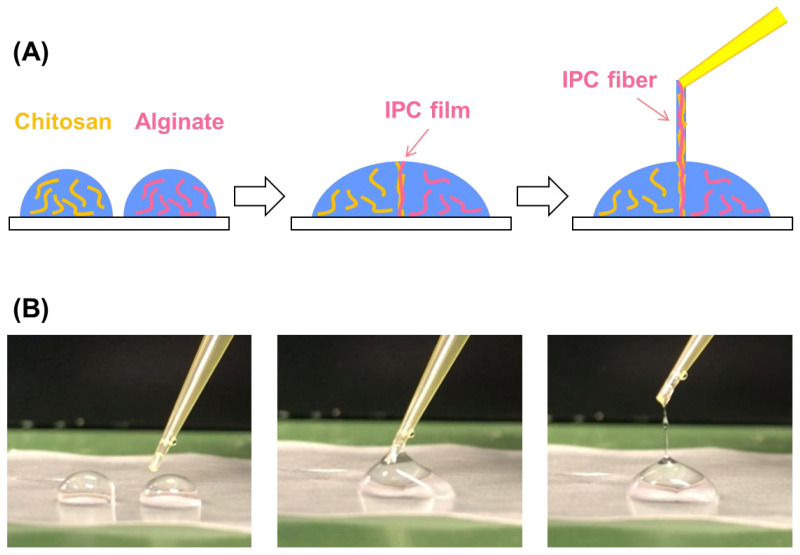
An IPC fiber of chitosan–alginate: (**A**) fabrication scheme; (**B**) photographs of fabrication. Droplets of chitosan and sodium alginate solutions were placed in contact, and an IPC fiber was drawn out using the pipette tip.

Figure 1A shows a scheme of IPC fiber fabrication. Fibers with lengths greater than 500 mm were easily fabricated (Figure 1B), and the fiber diameter ranged from 100 to 1000 µm. Further, beaded shapes were formed because of the rapid hand-drawing rate. However, as shown previously, beadless fibers can be fabricated by reducing the drawing rate [12]. In this study, beaded fibers were used because our focus was on the proof-of-concept of the electrochemical glue.

### 3.2. Electrochemical Gluing Induced by HClO

**Figure 2:**
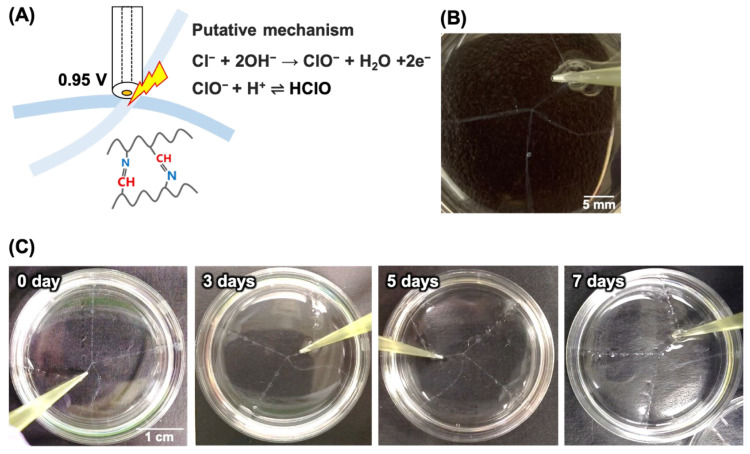
Electrochemical gluing via HClO: (**A**) Schematic. (**B**) Photograph of electrochemically glued hydrogel fibers. The fibers were floated in PBS, and the Cl^−^ concentration was adjusted to 0.30 M. Then, a voltage of 0.95 V vs. Ag/AgCl was applied to the intersection of the fibers for 300 s. (**C**) Time-course images of electrochemically glued IPC fibers in normal PBS for 7 days. Voltage application time: 900 s; Cl^−^ concentration: 0.50 M. The contrast and the brightness of the images were adjusted to improve fiber visibility.

Figure 2A shows a schematic of the electrochemical glue induced by HClO. Gray et al. reported a putative mechanism for the reaction of chitosan near the anode [41], and constant currents were applied to oxidize Cl^−^ to Cl_2_, resulting in the generation of HClO. In our study, a constant voltage of 0.95 V was selected, but this might be insufficient for oxidation. Therefore, we proposed another putative mechanism, as shown in Figure 2A. In our experiments, when 0.95 V was applied to a 0.30 M Cl^−^ solution, the hydrogel fibers were successfully glued together (Figure 2B), indicating that the formation of Schiff base linkages between the chitosan. In contrast, when a potential was not applied, no bonding occurred (Video S1), which suggests the crucial role of the microelectrode in bonding the hydrogel fibers. After incubation in PBS for 7 days, the crosslinked fibers remained intact (Figure 2C), indicating that the reactions were irreversible and no hydrolysis occurred during this period. Next, we investigated the effects of the chloride concentration and voltage application period on the electrochemical glue (Table 1). When using 0.15 M Cl^−^, the hydrogel fibers were hardly glued together, even with an increase in the voltage application time, suggesting that a 0.15 M Cl^−^ solution generated insufficient HClO to induce gluing. In contrast, when using 0.30 M Cl^−^, gluing was achieved after 3 min voltage application. Furthermore, the glue success rate (successful gluing/number of trials) was over 30% at 0.50 M, Cl^−^, even though the voltage was applied for only 1 min. Thus, the success rate increased as the chloride concentration and applied voltage time increased. Because chloride ions are common ions, this strategy is useful for assembling hydrogel pieces. However, under these experimental conditions, it was necessary to add further chloride to the D-PBS to achieve a high-concentration chloride solution, and these conditions might be unsuitable for cell culture. Therefore, to ensure successful cell culture, the sizes of the electrodes, electrode materials, and solution components should be optimized.

### 3.3. Electrochemical Gluing Induced by Ca^2+^

**Figure 3:**
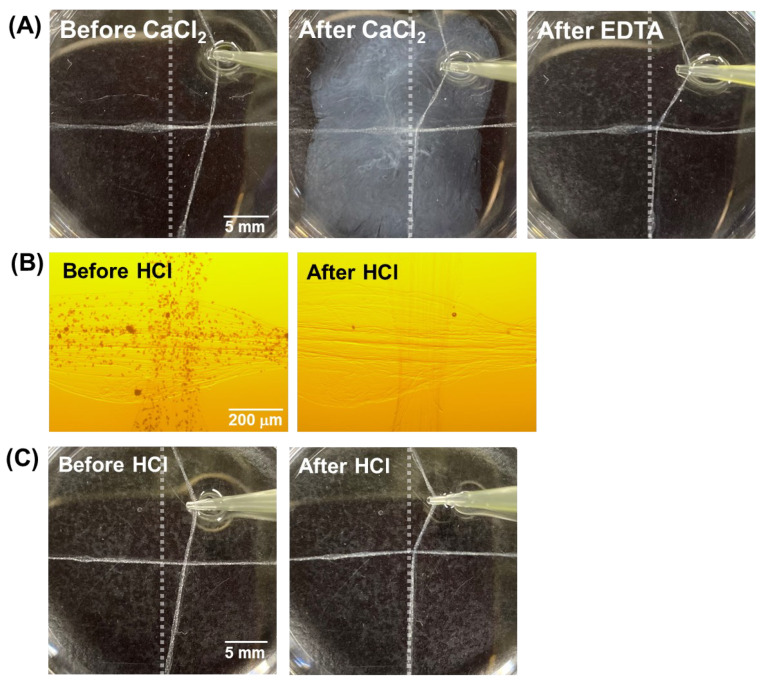
Binding of hydrogel fibers using CaCl_2_ or HCl solution. Two fibers were arranged orthogonally, and one of the fibers was dragged away from the other: (**A**) photographs of hydrogel fibers without CaCO_3_ particles before and after treatment with the CaCl_2_ solution; (**B**) microphotographs and (**C**) photographs of hydrogel fibers with CaCO_3_ particles before and after treatment with the HCl solution. Gray lines indicate the position of the fiber before dragging.

Next, we investigated the electrochemical generation of Ca^2+^ as a gluing agent. First, the effect of Ca^2+^ was investigated. Crucially, when CaCl_2_ solution was injected into the solution containing the hydrogel fibers, they became crosslinked owing to the interaction between Ca^2+^ and alginate. Subsequently, EDTA solution was injected into the glued area to chelate Ca^2+^ and remove it from the glued points. Surprisingly, even after EDTA treatment, the hydrogel fibers did not separate from another one, indicating that the alginate had formed interactions with the chitosan between the hydrogel fibers after gluing (Figure 3A).

Next, hydrogel fibers containing CaCO_3_ particles were fabricated. The particles were uniformly dispersed in the hydrogel fibers and disappeared after HCl had been added (Figure 3B), indicating that Ca^2+^ was released from the CaCO_3_ particles as a result of acidification. Next, the HCl solution was injected at the points where the hydrogel fibers containing CaCO_3_ particles crossed, resulting in their crosslinking (Figure 3C). This result indicated that Ca^2+^ released from CaCO_3_ particles after acidification acted as the glue for chitosan–alginate hydrogel fibers.

**Figure 4:**
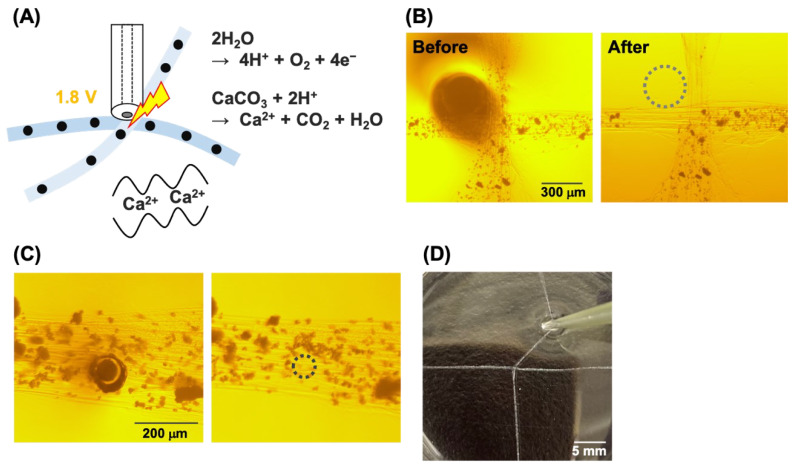
Electrochemical gluing via Ca^2+^: (**A**) Schematic. Black circles indicate CaCO_3_ particles. Microphotographs of hydrogel fibers before and after the application of 1.8 V using (**B**) the 300-µm-diameter Pt wire and (**C**) the 50-μm-diameter Pt disk microelectrode. (**D**) Photograph of electrochemically glued hydrogel fibers.

Next, we investigated whether electrochemically generated Ca^2+^ could be used as the glue. As shown in Figure 4A, a voltage of 1.8 V was applied to generate H^+^ at the electrode. Subsequently, the H^+^ dissolved the CaCO_3_ particles, which released Ca^2+^. Because a Pt electrode was used instead of an Au electrode, HClO was not generated. As a result, the alginate was crosslinked between the hydrogel fibers because of the presence of Ca^2+^. First, a Pt wire with a diameter of 300 µm was used to investigate the areas of acidification. As shown in Figure 4B, the CaCO_3_ particles disappeared after acidification of 300 µm from the electrode edges. In contrast, when using the Pt disk microelectrode with a 50 µm diameter, the CaCO_3_ particles were most likely dissolved only under the electrode (Figure 4C), because the electrode area was significantly smaller than that of the Pt wire. This result suggests that the use of a microelectrode is beneficial for local electrochemical gluing. Therefore, in the following experiments, a Pt disk microelectrode was used, and a voltage of 1.8 V was applied. However, bubbles were generated at this voltage, and the bubbles moved some of the CaCO_3_ particles. In addition, the generation of bubbles might cause pH changes by mixing the solution. For future applications, the applied potential should be further optimized. Despite the bubbles and as shown in Figure 4D, the hydrogel fibers were successfully glued at the crossing point. As shown in Figure 3A, treatment with EDTA did not result in the detachment of the hydrogel fibers from each other, and the bonding remained intact for at least 60 min. Thus, this result suggests that the electrochemical gluing using Ca^2+^ is also irreversible, as observed using HClO as well.

The hydrogel fibers could be attached by pushing them forcibly together because of their stickiness and electrostatic interactions. However, this physical approach could damage cells embedded in the hydrogels. In contrast, the electrochemical method does not require physical contact, and the generated chemicals quickly diffuse after the voltage application is stopped, suggesting that the electrochemical approach is superior to the physical approach.

Unfortunately, the quantitative analysis of the mechanical strength of the bonds was difficult due to the non-uniformity of the fibers. For quantitative analysis, the manual operation is unsuitable, and machine operation is required.

### 3.4. Electrochemical Gluing Induced by Ca^2+^ of Hydrogel Fibers Containing Cells

**Figure 5:**
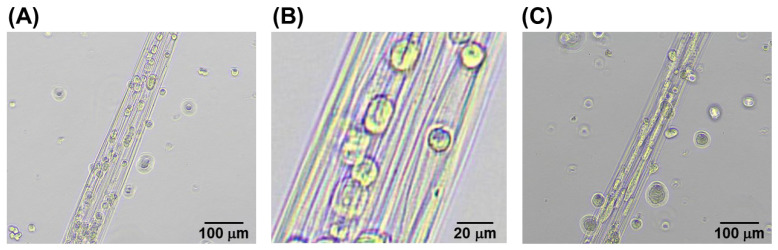
Cell culture in the hydrogel fibers after (**A**,**B**) 0 and (**C**) 2 days. (**B**) Magnified image. MCF-7 cells were incorporated into the hydrogel fibers, and these fibers were embedded in Matrigel.

Before investigating the cell culture after the electrochemical gluing process, we investigated the influence of the fabrication of IPC fibers on cell culture using the MCF-7 cell line. As shown in Figure 5A, the cells were uniformly incorporated into the hydrogel fibers, and the fiber structures remained after being embedded in Matrigel. Interestingly, some of the cells had become slightly elongated along the fiber at the end of the first day (day 0) (Figure 5B), indicating that gel fabrication affected the cell shape. After 2 days of culture, the MCF-7 cells had proliferated and formed cell fibers in the Matrigel (Figure 5C). Interestingly, the cell fibers were arranged along the direction of the hydrogel fibers. Thus, the fabrication process did not significantly affect the cell viability and enabled control over the shapes of cells and cell aggregates.

**Figure 6:**
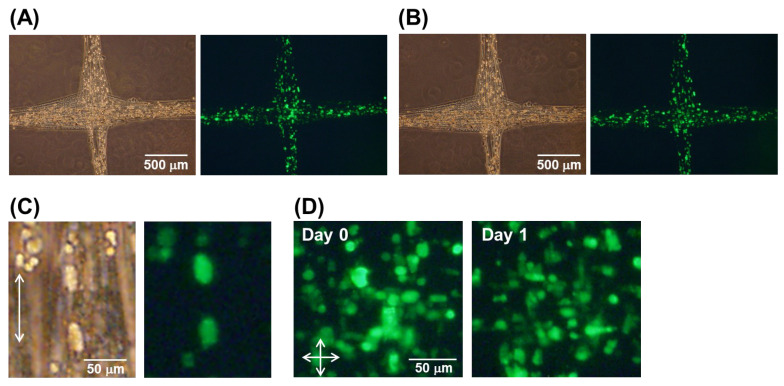
HUVECs embedded into hydrogel fibers after electrochemical gluing via Ca^2+^. The hydrogel fibers were cultured in culture medium. Phase-contrast and fluorescence images of the HUVECs after (**A**) 0 and (**B**) 1 day. (**C**) Magnified phase-contrast and fluorescence images after 0 days. The arrow indicates the direction of the hydrogel fibers. (**D**) Magnified fluorescence images at the crossing point after day 0 and day 1. Arrows indicate the direction of the hydrogel fibers.

Next, we investigated the cell culture of HUVECs after the application of the electrochemical glue. First, electrochemically glued hydrogel fibers containing HUVECs were grown in a culture medium. The HUVECs were also uniformly incorporated into the hydrogel fibers, and the electrochemical glue did not disturb their dispersion (Figure 6A). Further, although a voltage of 1.8 V was applied and acidification occurred, the cell morphology was not significantly changed. After 1 day of culture, the bond at the crossing points remained intact, indicating that the cell culture did not disturb the crosslinking (Figure 6B). As for the MCF-7 cells, some of the HUVECs elongated slightly along the direction of the hydrogel fibers (Figure 6C). The control of the cell shape may be useful for vascular formation in future applications. In addition, after 1 day of culture, the cells adhered to the hydrogel fibers further elongated at the electrochemically glued point (Figure 6D), indicating that this bonding method did not cause significant cell damage. In contrast, unlike MCF-7 cells, the HUVECs did not form cell aggregates because they are endothelial cells. Additionally, the cells did not grow well in the culture medium. Finally, the HUVECs did not form capillary structures or induce tube formation. Therefore, we embedded and cultured the cells in Matrigel.

**Figure 7:**
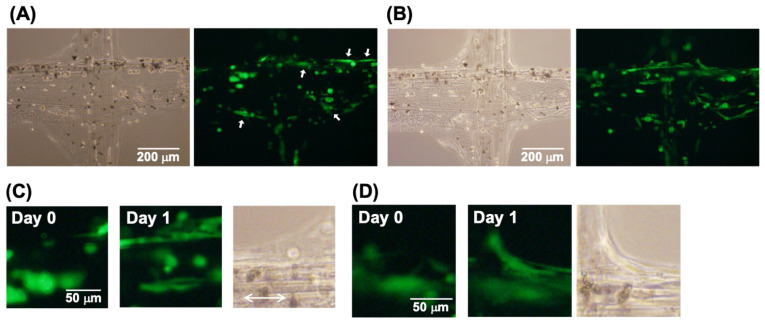
HUVECs embedded in hydrogel fibers after electrochemical gluing via Ca^2+^. The hydrogel fibers were cultured in Matrigel. Phase-contrast and fluorescence images of the HUVECs after (**A**) 0 and (**B**) 1 day. White arrows in (**A**) indicate cell adhesion. (**C**) Magnified fluorescence images after day 0 and day 1. An arrow indicates the direction of the hydrogel fibers. (**D**) Magnified fluorescence images at the crossing point after day 0 and day 1.

Figure 7 shows the culture of the electrochemically glued hydrogel fibers in Matrigel. Figure 7A shows the results obtained on the first day (day 0); the HUVECs adhered to the Matrigel and elongated during gelation. After 1 day of culture, the HUVECs formed capillary structures at the electrochemically glued points (Figure 7B), indicating that even after electrochemical gluing, the cells could be cultured in the Matrigel. In addition, the direction of the growing capillary followed the direction of the hydrogel fibers (Figure 7C). In contrast, the HUVECs spread on the hydrogel fibers at the crossing points bridged the two orthogonally arranged hydrogel fibers (Figure 7D). Thus, an electrochemically glued hydrogel structure can be used as a scaffold for HUVECs to control the cell growth direction. As a result, the cells grew much better than in the free medium.

## 4. Conclusions

In this study, we developed a novel strategy for the electrochemical bonding of chitosan–alginate hydrogel fibers. Electrochemically generated HClO and Ca^2+^ were used to crosslink chitosan via Schiff base formation and alginate via Ca^2+^ interactions, resulting in the binding of two hydrogel fibers. Next, we used the Ca^2+^-based electrochemical glue for cell culture. Embedded HUVECs proliferated and spread in the glued hydrogel fibers, demonstrating the effectiveness of the hydrogel for cell culture. Tube-like HUVEC structures were not formed, but this is the first report of electrochemical glue; thus, our study focused on the proof-of-concept of the electrochemical gluing process. Crucially, in the gluing, although HClO and H^+^ are formed, which lower the pH and affect cell viability, these chemicals diffuse through the gel and disappear once the applied voltage is stopped, resulting in low toxicity. Therefore, the on-demand generation approach is superior to other approaches for cell culture. We believe that the electrochemical glue concept will contribute to developments in tissue engineering and the fabrication of organs. Although we used fiber hydrogels as small construction units in the present study, this technique can be applied to other units such as beads and sheets. By assembling these units using this technique, more complex 3D hydrogels will be fabricated. However, for this purpose, it is necessary to characterize electrochemically glued hydrogels more deeply from the viewpoint of cell culture.

## Figures and Tables

**Table 1:** Success rate (number of successes/number of trials) of the electrochemical glue under various conditions. Red figures indicate that the success rate was over 50%.

Applied Time (min)	Cl^−^ Concentration		
	0.15 M	0.30 M	0.50 M
1	0/3	0/3	2/6
2	0/3	0/3	2/10
3	0/3	1/4	3/6
5	1/4	3/3	3/4
10	0/3	3/3	3/3
15	0/3	2/3	3/3

## Supplementary Materials

The following are available online at https://www.mdpi.com/article/10.3390/mi13030420/s1, Video S1: Electrochemically glued hydrogel fibers. Electrochemical glue was used to bind two hydrogel fibers via HClO. At the right cross points, 0.95 V was applied. At the left cross point, no potential was applied. Voltage application time: 900 s; Cl- concentration: 0.50 M.
